# Supplementary material for: The Defensive Activation Theory: REM Sleep as a Mechanism to Prevent Takeover of the Visual Cortex
Source: Front Neurosci. 2021 May 21;15:632853. doi: 10.3389/fnins.2021.632853 (PMC8176926; doi:10.3389/fnins.2021.632853)
Supplement: Supplementary file 1 [file Table_1.DOCX]

**The Defensive Activation theory: dreaming as a mechanism to prevent takeover of the visual cortex**

Supplemental Online Materials

### Supplemental Methods

We collected published measures of REM sleep (specifically, the fraction of sleep time spent in REM) for 25 species of primate, all of whom such measures were available: mongoose lemur [(1, 2)](https://paperpile.com/c/Pt9uGv/Eydp7+jXCsB), grey mouse lemur [(1, 3, 4)](https://paperpile.com/c/Pt9uGv/6bjht+Eydp7+Y4LaZ), vervet monkey [(1, 4, 5)](https://paperpile.com/c/Pt9uGv/gM032+Eydp7+Y4LaZ), patas monkey [(1, 5, 6)](https://paperpile.com/c/Pt9uGv/gM032+tCUMp+Eydp7), black lemur [(2, 4)](https://paperpile.com/c/Pt9uGv/jXCsB+Y4LaZ), barbary macaque [(1, 4, 7)](https://paperpile.com/c/Pt9uGv/o1Jh5+Eydp7+Y4LaZ), pigtail macaque [(8–10)](https://paperpile.com/c/Pt9uGv/bZc5S+gDVL8+9Beb5), guinea baboon [(11–13)](https://paperpile.com/c/Pt9uGv/9RtJY+WwpmS+ifgeE), three-striped night monkey [(1, 6, 14)](https://paperpile.com/c/Pt9uGv/TsyAZ+tCUMp+Eydp7/?locator_label=volume,page,page), bonnet macaque [(15–17)](https://paperpile.com/c/Pt9uGv/eutsA+7kgj6+bz5EH), bornean orangutan [(18)](https://paperpile.com/c/Pt9uGv/N5ZRU), hamadryas baboon [(4, 19)](https://paperpile.com/c/Pt9uGv/Gwhmg+Y4LaZ), olive baboon [(1, 20, 21)](https://paperpile.com/c/Pt9uGv/WV9UW+Eydp7+CCJnw), chimpanzee [(6, 22, 23)](https://paperpile.com/c/Pt9uGv/1pByk+gnHqQ+tCUMp), stump-tailed macaque [(1, 4, 24)](https://paperpile.com/c/Pt9uGv/g0SVK+Eydp7+Y4LaZ), long-tailed macaque [(25)](https://paperpile.com/c/Pt9uGv/i0KDn), common marmoset [(1, 25, 26)](https://paperpile.com/c/Pt9uGv/xO7m9+i0KDn+Eydp7), common squirrel monkey [(27–29)](https://paperpile.com/c/Pt9uGv/SPCJr+Ujm6U+WJoRi), rhesus monkey [(13, 30, 31)](https://paperpile.com/c/Pt9uGv/out2N+ifgeE+Gk2ub), human [(32–34)](https://paperpile.com/c/Pt9uGv/wX5b8+OAjAM+qZI2B), senegal galago [(4, 6, 35)](https://paperpile.com/c/Pt9uGv/CWT2o+tCUMp+Y4LaZ), yellow baboon [(4, 36)](https://paperpile.com/c/Pt9uGv/Y4LaZ+nAVN1), green monkey [(5, 37)](https://paperpile.com/c/Pt9uGv/gM032+nyy7p), spider monkey [(38)](https://paperpile.com/c/Pt9uGv/rRqwb), and brown lemur [(2)](https://paperpile.com/c/Pt9uGv/jXCsB).

### Supplemental Results

We gathered 4 variables not related to plasticity that were obtainable across these 25 primate species: body mass, length of body, number of offspring, and average lifespan.


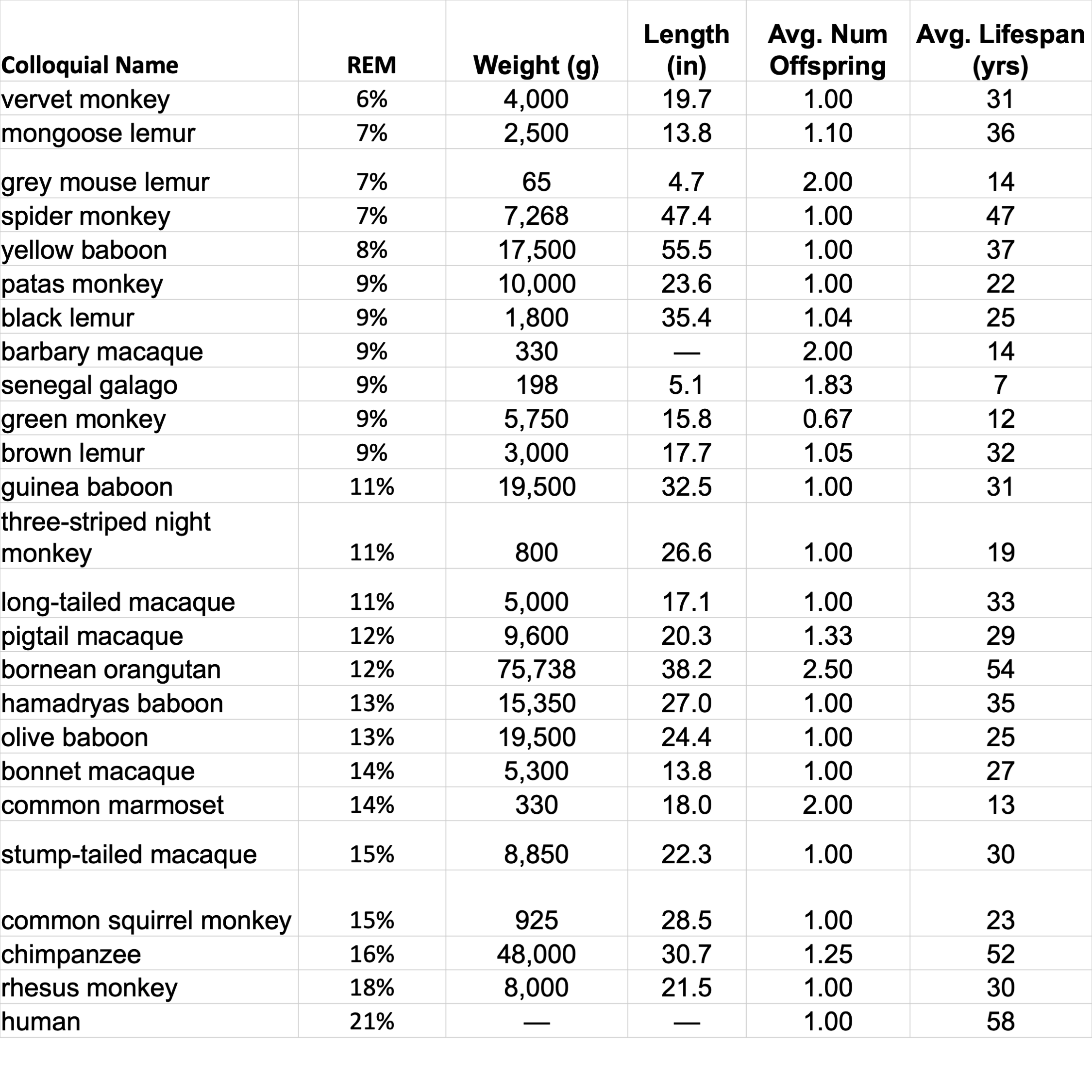


### References

1. [C. L. Nunn, P. McNamara, I. Capellini, P. Preston, R. A. Barton, Primate sleep in phylogenetic perspective. *Evolution of sleep: Phylogenetic and functional perspectives*, 123–144 (2010).](http://paperpile.com/b/Pt9uGv/Eydp7)

2. [E. Balzamo, G. Vuillon-Cacciuttolo, J. J. Petter, J. Bert, Etats de vigilance chez deux Lemuridae: rythmes EEG et organization obtenus par télémesure. *Sleeping Waking* **2**, 237–245 (1978).](http://paperpile.com/b/Pt9uGv/jXCsB)

3. [V. Barre, A. Petter-Rousseaux, Seasonal variations in sleep-wake cycle inMicrocebus murinus. *Primates* **29**, 53–64 (1988).](http://paperpile.com/b/Pt9uGv/6bjht)

4. [A. A. Gonfalone, S. K. Jha, The influence of gravity on REM sleep. *Open Access Anim. Physiol.* **7**, 65–72 (2015).](http://paperpile.com/b/Pt9uGv/Y4LaZ)

5. [J. Bert, V. Pegram, [The sleep electroencephalogram in Cercopithecinae: Erythrocerbus patas and Cercopithecus aethiops sabaeus]. *Folia Primatol.*  **11**, 151–159 (1969).](http://paperpile.com/b/Pt9uGv/gM032)

6. [H. Zepelin, A. Rechtschaffen, Mammalian Sleep, Longevity, and Energy Metabolism; pp. 447--470. *Brain Behav. Evol.* **10**, 447–470 (1974).](http://paperpile.com/b/Pt9uGv/tCUMp)

7. [F. F. González, J. Zaplana, C. Ruiz de Elvira, J. M. Delgado, Nocturnal and diurnal sleep in Macaca sylvana. *Electroencephalogr. Clin. Neurophysiol.* **46**, 13–28 (1979).](http://paperpile.com/b/Pt9uGv/o1Jh5)

8. [M. L. Reite, J. M. Rhodes, E. Kavan, W. R. Adey, NORMAL SLEEP PATTERNS IN MACAQUE MONKEY. *Arch. Neurol.* **12**, 133–144 (1965).](http://paperpile.com/b/Pt9uGv/bZc5S)

9. [K. Kaemingk, M. Reite, Social environment and nocturnal sleep: studies in peer-reared monkeys. *Sleep* **10**, 542–550 (1987).](http://paperpile.com/b/Pt9uGv/gDVL8)

10. [C. Batini, M. Radulovacki, R. T. Kado, W. R. Adey, Effect of interhemispheric transection on the EEG patterns in sleep and wakefulness in monkeys. *Electroencephalogr. Clin. Neurophysiol.* **22**, 101–112 (1967).](http://paperpile.com/b/Pt9uGv/9Beb5)

11. [J. Bert, E. Balzamo, M. Chase, V. Pegram, The sleep of the baboon, Papio papio, under natural conditions and in the laboratory. *Electroencephalogr. Clin. Neurophysiol.* **39**, 657–662 (1975).](http://paperpile.com/b/Pt9uGv/9RtJY)

12. [J. Bert, H. Collomb, L’électroencéphalogramme du sommeil nocturne chez le babouin. Etude par télémétrie. *J. Physiol. Paris* **58**, 285–301 (1966).](http://paperpile.com/b/Pt9uGv/WwpmS)

13. [J. Bert, V. Pegram, J. M. Rhodes, E. Balzano, R. Naquet, A comparative sleep study of two Cercopithecinae. *Electroencephalogr. Clin. Neurophysiol.* **28**, 32–40 (1970).](http://paperpile.com/b/Pt9uGv/ifgeE)

14. [A. A. Perachio, Sleep in the nocturnal primate, Aotus trivirgatus (1971) (January 2, 2020).](http://paperpile.com/b/Pt9uGv/TsyAZ)

15. [J. Bert, V. Pegram, E. Balzano, Comparaison du sommeil de deux macaques (Macaca radiata et Macaca mulatta). *Folia Primatol.*  **17**, 202–208 (1972).](http://paperpile.com/b/Pt9uGv/eutsA)

16. [J. Bert, Adaptation Du Sommeil Aux Conditions Experimentales D�enregistrement Chez Deux Cercopithecinae (Papio Papio Et Macaca Radiata). *Proceeding Of 3rd International Congress On Primatology* **2**, 49–53 (1970).](http://paperpile.com/b/Pt9uGv/7kgj6)

17. [M. Reite, R. Short, Behavior and physiology in young bonnet monkeys. *Dev. Psychobiol.* **19**, 567–579 (1986).](http://paperpile.com/b/Pt9uGv/bz5EH)

18. [C. L. Nunn, D. R. Samson, Sleep in a comparative context: Investigating how human sleep differs from sleep in other primates. *Am. J. Phys. Anthropol.* **166**, 601–612 (2018).](http://paperpile.com/b/Pt9uGv/N5ZRU)

19. [J. Bert, Similitudes et differences du sommeil chez deux Babouins, Papio Hamadryas et Papio Papio. *Electroencephalogr. Clin. Neurophysiol.* **35**, 209–212 (1973).](http://paperpile.com/b/Pt9uGv/Gwhmg)

20. [E. Balzamo, J. Bert, Sleep in Papio anubis: its organisation and lateral geniculate spikes. *Sleep Res. Online* **4**, 138 (1975).](http://paperpile.com/b/Pt9uGv/WV9UW)

21. [M. A. Elgar, M. D. Pagel, P. H. Harvey, Sleep in mammals. *Anim. Behav.* **36**, 1407–1419 (1988).](http://paperpile.com/b/Pt9uGv/CCJnw)

22. [E. Balzamo, R. J. Bradley, J. M. Rhodes, Sleep ontogeny in the chimpanzee: from two months to forty-one months. *Electroencephalogr. Clin. Neurophysiol.* **33**, 47–60 (1972).](http://paperpile.com/b/Pt9uGv/1pByk)

23. [J. Bert, D. F. Kripke, J. Rhodes, Electroencephalogram of the mature chimpanzee: twenty-four hour recordings. *Electroencephalogr. Clin. Neurophysiol.* **28**, 368–373 (1970).](http://paperpile.com/b/Pt9uGv/gnHqQ)

24. [L. Leinonen, D. Stenberg, Sleep in Macaca arctoides and the effects of prazosin. *Physiol. Behav.* **37**, 199–202 (1986).](http://paperpile.com/b/Pt9uGv/g0SVK)

25. [A. Ishikawa, *et al.*, Investigation of sleep-wake rhythm in non-human primates without restraint during data collection. *Exp. Anim.* **66**, 51–60 (2017).](http://paperpile.com/b/Pt9uGv/i0KDn)

26. [H. S. Crofts, *et al.*, Investigation of the sleep electrocorticogram of the common marmoset (Callithrix jacchus) using radiotelemetry. *Clin. Neurophysiol.* **112**, 2265–2273 (2001).](http://paperpile.com/b/Pt9uGv/xO7m9)

27. [D. B. Wexler, M. C. Moore-Ede, Circadian sleep-wake cycle organization in squirrel monkeys. *Am. J. Physiol.* **248**, R353–62 (1985).](http://paperpile.com/b/Pt9uGv/SPCJr)

28. [P. M. Adams, E. S. Barratt, Nocturnal sleep in squirrel monkeys. *Electroencephalogr. Clin. Neurophysiol.* **36**, 201–204 (1974).](http://paperpile.com/b/Pt9uGv/Ujm6U)

29. [P. Breton, P. Gourmelon, L. Court, New findings on sleep stage organization in squirrel monkeys. *Electroencephalogr. Clin. Neurophysiol.* **64**, 563–567 (1986).](http://paperpile.com/b/Pt9uGv/WJoRi)

30. [E. Balzamo, P. Van Beers, D. Lagarde, Scoring of sleep and wakefulness by behavioral analysis from video recordings in rhesus monkeys: comparison with conventional EEG analysis. *Electroencephalogr. Clin. Neurophysiol.* **106**, 206–212 (1998).](http://paperpile.com/b/Pt9uGv/out2N)

31. [Robinson, E. L., Hsieh, J. K., & Fuller, C. A., A Primate Model Of Sleep Regulation [Abstract]. *Sleep* **26 (Suppl.)**, A391 (2003).](http://paperpile.com/b/Pt9uGv/Gk2ub)

32. [M. A. Carskadon, W. C. Dement, Normal Human Sleep: An Overview. *Principles and Practice of Sleep Medicine*, 13–23 (2005).](http://paperpile.com/b/Pt9uGv/wX5b8)

33. [J. A. Floyd, J. J. Janisse, E. S. Jenuwine, J. W. Ager, Changes in REM-sleep percentage over the adult lifespan. *Sleep* **30**, 829–836 (2007).](http://paperpile.com/b/Pt9uGv/OAjAM)

34. [H. P. Landolt, D. J. Dijk, P. Achermann, A. A. Borbély, Effect of age on the sleep EEG: slow-wave activity and spindle frequency activity in young and middle-aged men. *Brain Res.* **738**, 205–212 (1996).](http://paperpile.com/b/Pt9uGv/qZI2B)

35. [J. Bert, H. Collomb, A. Martino, L’électroencéphalogramme de sommeil d'un pro-simien. Sa place dans l'organisation du sommeil chez les primates. *Electroencephalography and Clinical Neurophysiology* **23**, 342–350 (1967).](http://paperpile.com/b/Pt9uGv/CWT2o)

36. [E. Balzamo, Etude Des Etats De Vigilance Chez Papio Cynocephalus Adulte. *C. R. Seances Soc. Biol. Fil.* **167**, 1168–1172 (1973).](http://paperpile.com/b/Pt9uGv/nAVN1)

37. [Balzamo, E., Vuillon-Cacciuttolo, G., & Bert, J., Cercopithecus Aethiops: EEG Et Organization Des Etats De Vigiliance. *Wake Sleep* **2**, 223–230 (1978).](http://paperpile.com/b/Pt9uGv/nyy7p)

38. [M. A. Cruz-Aguilar, *et al.*, Sleep in the spider monkey (Ateles geoffroyi): A semi-restrictive, non-invasive, polysomnographic study. *Am. J. Primatol.* **77**, 200–210 (2015).](http://paperpile.com/b/Pt9uGv/rRqwb)
